# Effect of intrapartum fetal stress associated with obstetrical interventions on viability and survivability of canine neonates

**DOI:** 10.14202/vetworld.2016.1485-1488

**Published:** 2016-12-30

**Authors:** Karthik V. Kuttan, Metilda Joseph, Shibu Simon, K. N. Aravinda Ghosh, Anish Rajan

**Affiliations:** Department of Animal Reproduction Gynaecology and Obstetrics, College of Veterinary and Animal Sciences, Mannuthy, Thrissur - 680 651, Kerala, India

**Keywords:** canine neonates, neonatal stress, umbilical lactate

## Abstract

**Aim:**

This study was conducted with the objective of identifying and evaluating intrapartum fetal stress in connection with the type of delivery in bitches.

**Materials and Methods:**

A total of 26 bitches between 1 and 5 years, belonging to 10 different breeds were evaluated. Bitches were subjected to detailed clinico-gynecological examination based on history. Neonatal stress associated with spontaneous whelping (SW), assisted whelping (AW), and emergency cesarean section (EC) was evaluated using umbilical vein lactate (UL) estimation by collecting the blood from umbilical vein.

**Results:**

A high umbilical vein lactate value was associated with fetal distress. The mean umbilical lactate value was highest in EC (12.54±0.8 mmol/L) followed by AW (8.86±0.9 mmol/L) and the lowest value was found in SW (7.56±0.58 mmol/L). A significant increase (p<0.05) in umbilical lactate level was observed in EC group of canine neonates compared with AW and SW groups. Overall mean umbilical lactate values of neonates which died within 24 h (13.31±1.08 mmol/L) and the neonates which survived beyond 24 h (8.87±0.55 mmol/L) differed significantly at 5% level.

**Conclusion:**

Immediate identification of neonatal distress by use of umbilical vein lactate estimation is helpful for the clinician to undertake resuscitation or medical therapy to ensure better neonatal survivability.

## Introduction

Canine breeding is one of the fastest growing animal husbandry enterprise considered as an important source of income for many unemployed youth in Kerala. Parturition is one of the most anxious time for dog breeders, as the survival chances of the puppy and the dam are influenced by management techniques and clinical interventions made at this stage. Further, increased financial and emotional value of stud dogs, brood bitches and their offspring to the pet fancy makes the preventable loss of even one neonate undesirable [1].

Canine pregnancies are unique among veterinary domestic species as the whelping process is longer in duration and the neonates are highly influenced by the environmental factors [2]. Labor represents the most critical phase for the neonates and the stress associated with parturition contributes a major share to the neonatal mortality which is reported to be between 5% and 35% in canines [3]. Perinatal mortality is influenced by the canine breed, litter size, and maternal age [4]. Lactate plays an important role in human obstetrics as a marker of neonatal distress [5]. The presence of higher level of lactate in umbilical cord blood suggests the use of secondary oxygenation pathways due to hypoxic events occurring during parturition [6]. Only a little quota of lactate cross the placenta, so lactic acid in fetal blood during labor is thought to be primarily of fetal origin [7,8] and is the end product of anaerobic glycolysis [8]. It was identified that 5 mmol/L of umbilical vein lactate concentration could be used as the cut off value, to distinguish between healthy and distressed pups. Thus, sampling of the blood from the umbilical cord during parturition and estimation of the lactate in the cord blood will help the clinician in easy and timely identification of the distressed neonates. Timely intervention is always necessary to improve neonatal outcome and so the techniques/procedures for the identification of neonatal distress should be simple and less time consuming [9]. Prudent veterinary intervention in the prenatal, parturient, and postpartum periods can increase neonatal survival by controlling the factors contributing to neonatal morbidity and mortality [10].

## Materials and Methods

### Ethical approval

The experiments comply with the guidelines laid down by the Institutional Ethical Committee and in accordance with the country law.

### Study area

This study was conducted in Thrissur district, Kerala, India. 26 whelping cases consisting of bitches between one to five years of age and belonging to 10 different canine breeds, with clinical signs suggestive of an impending whelping were randomly selected for the study. Five spontaneous whelping (SW) cases with a total of nine puppies were examined out of which four whelpings were attended at breeder’s premises, and one bitch was presented to University Veterinary Hospital, Kokkalai. 13 assisted whelping (AW) cases with 13 puppies and eight emergency cesarean section (EC) cases consisting of 13 puppies were evaluated.

### AW protocol

Clinical interventions such as lubrication with obstetrical cream, feathering, traction by hand/whelping forceps were performed wherever necessary. In the case of uterine inertia, 10 per cent calcium gluconate solution at the rate of 0.2 ml/kg was administered intravenously along with 25% dextrose over a period of 2-5 min. Oxytocin was administered at the dose rate of 0.1-0.5 IU/kg intramuscularly and repeated after 60-90 min whenever necessary.

### Anesthetic regimen for EC section

Animal was premedicated with an intramuscular administration of atropine sulfate at the dose rate of 0.045 mg/kg body weight and xylazine hydrochloride at the dose rate of 1 mg/kg body weight. Anesthesia was induced by intramuscular injection of ketamine hydrochloride at the dose rate of 5 mg/kg body weight and intravenous administration of diazepam at the dose rate of 0.5 mg/kg. Anesthesia was maintained with intravenous administration of a mixture of xylazine and ketamine at 1:1 ratio and diazepam wherever necessary. Before cesarean section, antibiotics and fluid were administered intravenously [11].

### Umbilical vein lactate estimation in canine neonates

**Figure-1:**
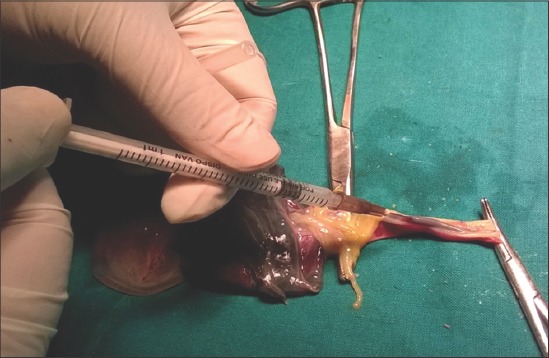
Collection of blood from umbilical vein of the neonate after doubly clamping the umbilical cord.

To estimate lactate level, blood sample was collected from the umbilical vein of the neonates. The umbilical cord was doubly clamped to isolate the blood vessels from the placenta [12]. A blood sample of about 25 µL was collected as soon as possible after birth using a tuberculin syringe with a 26 G needle (Figure-1).

One drop of blood sample was applied to the application area of the Accutrend^®^ Plus analyzer using a tuberculin syringe as soon as possible and never later than 5 min after birth. The Accutrend^®^ plus lactate technology was based on enzymatic determination and reflectance photometry (wavelength 660 nm) of lactate in a sample of whole blood. The measuring range is from 0.8 to 22 mmol/L.

### Statistical analysis

Records were enclosed in a database and statistically analyzed using the statistics software package SPSS (version 21).

## Results

### Umbilical vein lactate values

Mean umbilical lactate values of puppies born from different whelping groups are furnished in Table-1.

**Table-1:** Mean umbilical lactate value of the neonates according to the type of delivery.

Type of whelping	Number of bitches	Number of neonates (n)	Mean UL
AW	13	13	8.86±0.89^a^
Emergency	8	13	12.53±0.79^b^
C-section			
Normal whelping	5	9	7.55±0.57^a^
Total	26	35	9.81±0.58

Means with different superscript in columns differ significantly at 5% level

The umbilical lactate values in AW group, EC group and SW group were 8.86±0.89 (2.7-16.8) mmol/L, 12.53±0.79 (8.5-18.2) mmol/L and 7.55±0.57 (5.3-10.7) mmol/L, respectively. Average umbilical lactate value from all the groups was 9.81±0.58 mmol/L. A significant increase (p<0.05) in umbilical lactate value was observed in EC group of neonates.

### Associations of umbilical vein lactate with other parameters

Umbilical lactate values were negatively correlated with Apgar scoring (p<0.01) regardless of the type of delivery. High lactate concentrations were associated with low Apgar scores. Umbilical lactate values were positively correlated to temperature of puppies (p<0.01).

Correlation coefficients (r) of parameters of the bitch and neonates on umbilical lactate are furnished in Table-2. Umbilical lactate values were negatively correlated to heart rate (p<0.01) and respiratory rate (p<0.01) of puppies regardless of the type of delivery. Umbilical lactate values were negatively correlated with body weight of puppies (p<0.05) at birth. Umbilical lactate values were negatively correlated with litter size (p<0.05) of the dam.

**Table-2:** Correlation coefficients (r) of parameters of the bitch and neonates on umbilical lactate value.

Parameters	Umbilical lactate
Temperature of puppies	0.951**
Body weight of puppies	−0.393*
Body weight of the bitch	−0.276^ns^
Expulsion time	0.359^ns^
Age of the bitch	0.049^ns^
Litter size of the bitch	−0.362*
Duration of whelping	0.106^ns^

** Correlation is significant at 1% level;

* Correlation is significant at 5% level. ^ns^Correlation is nonsignificant

Puppies born in anterior presentation showed a lower mean umbilical lactate value of 7.8±0.69 mmol/L compared to those born in posterior presentation with a mean value of 10.2±0.45 mmol/L.

## Discussion

The high lactate value in AW could be due to fetal hypoxia developed through a prolonged expulsion time due to the late presentation of the bitch to hospital. The unscientific use of ecbolics like oxytocin by dog breeders caused uterine tetany and fetal hypoxia. The ecbolics did not have a direct effect on elevation of lactate but had an indirect effect possibly due to the impairment of the blood flow to the placenta, premature placental separation, and in utero fetal death [13]. In EC, the higher values may be due to the combined effect of prolonged expulsion time and the effects of ecbolics. In both AW and EC prolonged expulsion time contributed more toward the elevated lactate levels.

Umbilical lactate values were negatively correlated to heart rate (p<0.01) and respiratory rate (p<0.01) of puppies regardless of the type of delivery. This is in accordance with Schweizer and Meyers-Wallen [14] who reported that there is a significant correlation between fetal heart rate decelerations and fetal stress.

The overall mean umbilical lactate levels of dead and live puppies of all the whelping groups together were significantly different at 5% level (Table-3). This result indicates that the puppies that were dead were severely stressed due to hypoxia produced as a result of combined effect of anesthetics used in cesarean section and ecbolics used during AW [15].

**Table-3:** Effect of obstetrical interventions on viability of canine neonates.

Type of whelping	Number of bitches	Number of neonates	Mean±SE	

			Mean umbilical lactate of dead puppies (mmol/L)	Mean umbilical lactate of live puppies (mmol/L)
AW	13	13	10.95±0.75	8.48±1.02
EC	8	13	14.1±1.28	11.20±0.73
SW	5	9	-	7.55±0.57
Total	26	35	13.31±1.08^a^	8.87±0.55^b^

Means with different superscript in rows differ significantly at 5% level. AW=Assisted whelping, EC=Emergency cesarean section, SW=Spontaneous whelping, SE=Standard error

Out of 35 puppies evaluated, 7 were dead within 24 h which included 1 (7.6%) puppy from AW and 6 (46.15%) puppies from EC. None of the neonates evaluated in SW were dead within 24 h. This is in contrrary to the reports of Veronesi *et al*. [16] who reported that there is a higher risk of death in puppies born by AW than SW or cesarean section. The high mortality of puppies in emergency cesarean section in this study may be due to the influence of anesthetics such as ketamine, xylazine, and diazepam which caused fetal depression as described byMcDonnell and van Corder [17] who stated that xylazine rapidly crossed the placenta and induced both maternal and fetal respiratory and circulatory depression. Moon *et al*. [18] indicated that ketamine use lead to respiratory depression, apnea, decreased vocalization, and increased mortality at birth and diazepam caused severe fetal depression. Gowda [19] reported a neonatal survival rate of 96.67% when elective cesarean section was performed by induction with propofol and maintenance of anesthesia with isoflurane. The difference in the neonatal mortality in this study may be attributed to the emergency nature of cesarean section and the negative influence of the anesthetics on the fetus.

## Conclusion

From these results, it can be concluded that neonatal distress was more evident in puppies born through EC section and least distress was found in puppies born by normal delivery. Umbilical vein lactate estimation proved to be an easy and reliable technique for the prompt identification of neonatal distress. Such an early and prompt identification of neonatal distress will be useful in providing early neonatal care and resuscitation for the distressed neonates thereby reducing neonatal mortality.

## Authors’ Contributions

KVK designed and planned this research work, collected the samples and executed the entire work. MJ, SS and KNAG guided and monitored the research work. AR helped in sample collection and data analysis. All authors read and approved the final manuscript.
